# Magnetic Tactile Sensor with Bionic Hair Array for Sliding Sensing and Object Recognition

**DOI:** 10.1002/advs.202306832

**Published:** 2024-01-18

**Authors:** Jiandong Man, Zhenhu Jin, Jiamin Chen

**Affiliations:** ^1^ State Key Laboratory of Transducer Technology Aerospace Information Research Institute Chinese Academy of Sciences Beijing 100190 P. R. China; ^2^ School of Electronic Electrical and Communication Engineering University of Chinese Academy of Sciences Beijing 100049 P. R. China; ^3^ College of Materials Sciences and Opto‐Electronic Technology University of Chinese Academy of Sciences Beijing 100049 P. R. China

**Keywords:** flexible electronics, magnetic material, object recognition, sliding sensing, tactile sensor

## Abstract

Due to the high application value in intelligent robots, tactile sensors with large sensing area and multi‐dimensional sensing ability have attracted the attention of researchers in recent years. Inspired by bionics of hairs on human skin, a flexible tactile sensor based on magnetic cilia array is developed, showing extremely high sensitivity and stability. The upper layers of the sensor are multiple magnetic cilia containing magnetic particles, while the lower layer is a serpentine flexible circuit board with a magnetic sensor array. When magnetic cilia are bent under force, the magnetic sensor array can detect changes in the magnetic field, thereby the magnitude and direction of external force can be obtained. The proposed sensor has a resolution of 0.2 mN with a working range of 0–19.5 mN and can distinguish the direction of external force. The large sensing area and short response time make this sensor suitable for sliding tactile detection, and experiments show that the sensor can be also applied in object recognition with a success accuracy of 97%. In addition to the shape of objects, the sensor can identify whether there is magnetism inside objects, making it of significant value in intelligent robots and modern medicine.

## Graphical abstract

A biomimetic magnetic tactile sensor with a magnetic hair array is developed, which can accurately measure the magnitude of external forces with a remarkable resolution of 0.2 mN within a range of 0–19.5 mN. It excels in sliding sensing and object recognition with an accuracy rate of 97%. The exceptional performance makes this sensor highly suitable for intelligent robotics.

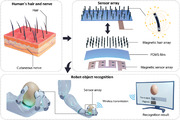

## 1 Introduction

Tactile sensation is one of the significant senses of humans, like vision and hearing.^[^ ^1^ ^]^ While the development of robot visual and auditory sensors has entered a commercialization stage, the development of tactile sensors is still immature.^[^ ^2^, ^3^ ^]^ This is due to the difficulty in achieving large‐area multi‐dimensional tactile perception. Tactile sensors based on piezoresistive,^[^ ^4^, ^5^ ^]^ piezoelectric,^[^ ^6^, ^7^ ^]^ capacitive,^[^ ^8^, ^9^ ^]^ and other principles^[^ ^10^ ^]^ have been widely studied. However, each kind of these sensors has some drawbacks. Piezoresistive tactile sensors are sensitive to temperature, so the devices have a large temperature drift. Piezoelectric tactile sensors need dynamic input, so they are not suitable for tactile force, which is mostly static. Tactile sensors based on capacitance are often affected by stray capacitance, and their output is unstable after prolonged use. Tactile sensing technology based on magnetic sensor has advantages of high resolution, low mechanical hysteresis, and non‐contact measurement, which is a crucial research direction for large‐scale application of tactile sensors in the future.^[^ ^11^, ^12^ ^]^ The basic principle of magnetic tactile sensors is using the mixture of magnetic material and flexible material as a magnetic field source. When the external force is applied on the mixture, the mixture will be deformed or displaced. The magnitude and direction of the external force can be obtained by detecting the change of the magnetic field with magnetic sensors nearby.^[^ ^11^ ^]^

In past researches, there have been many kinds of structures of the magnetic mixture.^[^ ^13^, ^14^, ^15^ ^]^ Based on the bionics of human skin, planar films with magnetism are used in most of studies.^[^ ^16^, ^17^ ^]^ However, this structure is more suitable for detecting normal force, but is not friendly to the perception of tangential force. In order to detect tangential force, some studies have used pyramid or arc shaped protrusion structures.^[^ ^7^, ^14^ ^]^ However, the sensitivity and resolution of sensors with film and protrusion structures are difficult to improve, and they are difficult to detect small force less than 1 mN.

**Figure 1:**
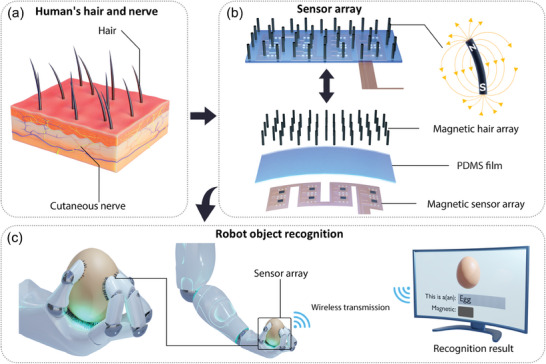
Hairs on human skin, structural diagram of our sensors, and sensor applications. a) Hairs on the human skin and the nerves under the skin. b) Schematic and exploded view of our sensor, with the right image showing the magnetic field distribution when a single cilium is bent. c) Schematic diagram of object recognition with our sensor. After the robotic arm with our sensor grabs an object, the recognition results and whether the object is magnetic can be displayed on a screen.

In fact, not only human skin itself has the ability of tactile perception, but also hairs on the skin. This is also very common in nature, such as hairs on spider legs and lateral structures of fish.^[^ ^18^ ^]^ The hair structure on human skin is shown in **Figure** **1**a. When hairs are bent by force, the nerve under hairs will transmit signals to human brain. Compared with membrane structures, ciliary structures can detect the tangential force and can greatly increase the sensitivity to small forces.^[^ ^19^, ^20^, ^21^ ^]^ Previously, we have conducted relevant researches with ciliary structures.^[^ ^22^ ^]^ However, in our previous research, only a single cilium is used to achieve a single point measurement, which cannot achieve large‐area tactile perception, and could not detect the sliding of objects. Therefore, it is difficult to apply it in fields that require large‐area perception, such as flexible grippers of robots. And it is also difficult to realize complex functions such as sliding detection and object recognition.

In order to solve these problems mentioned above, in this work, we propose a magnetic hair array. The structure is shown in Figure 1b, which is composed of upper, middle, and lower layers. The upper layer and the middle layer are a magnetic hair array and a flexible polydimethylsiloxane (PDMS) film respectively, while the lower layer is a flexible printed circuit (FPC) board with a magnetic sensor array. The number and area of magnetic cilia can be flexibly adjusted according to applications. The advantages of this work are as follows: 1) Our sensor can realize large‐area tactile perception, which makes it have the ability of sliding tactile perception. 2) Our sensor is able to detect tangential force and distinguish the direction of contact force. 3) When our sensor is applied in object recognition, in addition to the shape of the object, it can distinguish whether there is magnetism inside the object. These advantages prove that our sensor has great application potential in intelligent robots and modern medical treatment.

## 2 Experimental Section

### 2.1 Sensing Concept

The working principle of this sensor is that when the cilia array is bent by external force, the magnitude and direction of stray magnetic field under the cilia array will change. This change was detected by a magnetic sensor array on an FPC board. Then the magnitude and direction of external force can be obtained by algorithm processing. Finally, it can be known whether the object was sliding and its shape by the time sequence and size difference of signals of different sensors.

A single cilium was simplified to a cylindrical cantilever beam with a single fixed end for ease of understanding. When it was fixed at the bottom, the relationship between the force and the displacement of the top of the cantilever beam is shown in Equation (1)

(1) $$\delta =F\frac{64l^{3}}{3\pi ED^{4}}$$

where δ is the deflection distance of the top of the cylinder, *F* is the force acting on the top of the cylinder, *l* is the length of the cylinder, *E* is the Young's modulus of the material, and *D* is the diameter of the cylinder. It can be seen that the deflection distance was positively correlated with the force and the sensitivity of the sensor can be flexibly adjusted by changing the length, diameter, and Young's modulus of the cilium.

When magnetic cilia were bent under force, the stray magnetic field around them changed. Linear Hall sensors were used to detect this change and the relationship between Hall voltage and the size of external magnetic field is shown in Equation (2)

(2) $$V_{H}=\frac{I_{S}B}{ned}$$

where *n* and *d* are the carrier concentration and thickness of the Hall material, respectively. *e* is the electronic charge while *I*_S_is the current flowing through the Hall material. *n* and *d*are only related to the Hall material itself and *e* is a fixed value. When the working current *I_S_* is fixed, the Hall voltage *V_H_* is only positively related to the change in magnetic strength *B*. Therefore, the size of external force can be obtained by collecting *V*_H_.

### 2.2 Simulation

**Figure 2:**
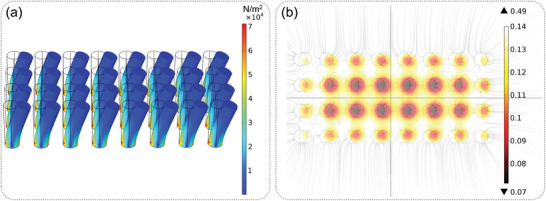
Simulation of our sensor. a) Simulation of stress and displacement when magnetic cilia are bent. b) Simulation of magnetic distribution of cilia array.

A finite element simulation was carried out to illustrate the distribution of stray magnetic field when magnetic cilia were bent. First, a solid mechanics component was used to simulate the deformation and stress distribution of cilia, as shown in **Figure** **2**a. The bottom of each cilium was set as a fixed constraint, and the rest was set as free. A boundary load was added to the side of the left half cylinder of each cilium to cause deformation of the cilium. This step was to obtain a deformed geometric model for subsequent magnetic simulations. Subsequently, the bent cilia model was saved and used to simulate the magnetic field distribution by setting the magnetic scalar potential at both ends of the cilium. The results are shown in Figure 2b. It can be seen that the magnetic field distribution shifts to the right as a whole as the cilia bend to the right. The number of cilia was very large, so placing one magnetic sensor under each cilium makes data processing very complex. For simplicity, a 2 × 4 array of magnetic sensors was placed under the cilia to detect magnetic changes. For an array, the magnetic field distribution was more complex than that of a single cilium. Therefore, in applications such as manipulator grasping, machine learning algorithms were used to help the authors with data processing.

### 2.3 Fabrication

**Figure 3:**
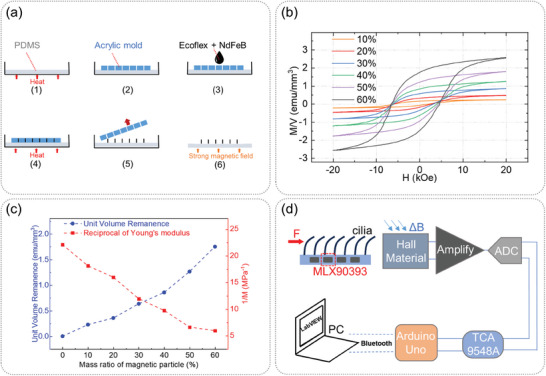
Production process and circuit design of our sensor. a) Production process of our sensor. b) VSM test results of mixtures with different magnetic particle contents. c) Unit volume remanence and Young's modulus of mixtures with different magnetic particle contents. d) Circuit design of our sensor.

Demolding method was used to make the magnetic cilia array. The manufacturing process is shown in **Figure** **3**a. First, PDMS (Sylgard 184 Silicone Elastomer, Dow Corning Corporation) solvent and curing agent were fully mixed in a rectangular container with a ratio of 10:1, and the thickness of PDMS was controlled to 1 mm. The PDMS film was used as the connecting layer of the magnetic cilia array. The thickness of 1 mm can ensure a strong connection of the cilia array and maintain an excellent flexibility. Second, the container was placed in a vacuum oven to remove bubbles for 20 min, and then placed in a baked oven at 80 °C for 5 min for incomplete curing. Third, A polymethyl methacrylate (PMMA) mold with 0.5 mm diameter circular holes made by laser cutting was placed on the top of the incompletely cured PDMS film. The purpose of incomplete curing was to ensure that the PMMA mold and PDMS film can be fully contacted without air gap. Then the container was placed in a baked oven at 80 °C and heated for 1 h for completely curing. Fourth, NdFeB magnetic particles (New NUODE, China) with an average diameter of 5 µm were fully mixed with solvent A and solvent B of Ecoflex (Smooth‐On, 0050) by using an electric rotary mixer (ZHEBEI, ZY3075) for 5 min. The mixture was poured over the PMMA mold and filled into the circular holes. Fifth, the container was placed in a vacuum oven to remove bubbles and then was put in the baked oven and heated at 80 °C for 1 h to cure completely. Sixth, the redundant solidified mixture on the surface of PMMA mold was removed by a knife. Then the PMMA mold was peeled off and a magnetic cilia array was left behind.

When fabricating cilia with a large aspect ratio, demolding poses a formidable challenge. The authors' investigation encompassed two primary facets: mold materials and cilia materials. Initially, glass and silicon wafers were employed as mold materials, with MEMS technology employed for etching and punching. But this requires thick glass or silicon wafers with a thickness of several millimeters, which was costly and takes a long production time. Although metal molds were also utilized, drilling small‐diameter holes in thick metal plates proved exceedingly intricate and expensive. Ultimately, PMMA, characterized by low cost and well‐established processing technology, was selected as the template. Subsequently, in the realm of cilia materials, PDMS was initially utilized. Nevertheless, the adhesion between PDMS and PMMA was pronounced, leading to the breakage of most cilia during demolding. This experiment found that Ecoflex exhibited low adhesion to PMMA, facilitating easy detachment from the mold. Consequently, Ecoflex emerged as the authors' ultimate choice. At the end of the production steps, the cilia array was magnetized vertically in a strong magnetic field with a size of 2 Tesla.

**Figure 4:**
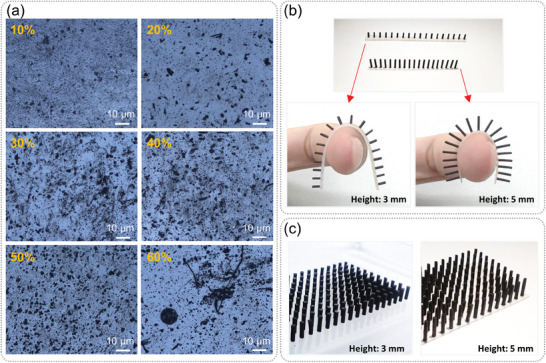
Microscopic images of mixtures with different magnetic particle contents and photos of cilia arrays made by demolding method. a) Microscopic images of mixtures with different magnetic particle contents. When the content exceeds 50%, magnetic particles aggregate and bubbles appear in mixtures. b) Single row magnetic cilia with different heights. c) Magnetic cilia arrays with different heights.

The mass ratio of magnetic particles to Ecoflex had a great influence on the magnetic properties of magnetic cilia. A physical property measurement system (Quantum Design, PPMS with VSM‐9T) was used to characterize the magnetic properties of magnetic cilia. The results are shown in Figure 3b. It can be seen that with the increase of the mass ratio of magnetic particles, the magnetism of cilia was increasing. According to Equation (2), increasing the magnetic particle content was of great help to improve the sensitivity of this sensor. However, as shown in Figure 3c, when the content of magnetic particles increases, the flexibility of cilia decreases (the right ordinate in Figure 3c was the reciprocal of Young's modulus). More importantly, with the increase of the content of magnetic particles, the manufacturing difficulty also increases. The magnetic particles were easy to aggregate in Ecoflex, and the uniformity of distribution was greatly reduced. Microscopic images of magnetic mixtures with different mass ratios are shown in **Figure** **4**a. It can be seen that when the content of magnetic particles reaches 60%, particles gather together in the mixture and the uniformity of the mixture was low. In addition, it was difficult to remove bubbles and bubbles appear on the mixture surface. For these reasons, finally a mass ratio of 50% was chosen to make the cilia array. A variety of PMMA molds with different array sizes were designed, including single row and multiple rows. The thicknesses of PMMA molds includes 3 and 5 mm. The cilia array with single row and multiple rows are shown in Figures 4b and 4c, respectively. They can be applied in different fields as required.

The schematic diagram of this cilia sensor is shown in Figure 3d. The magnetic sensors used in this work were three‐axis linear Hall sensors (MLX90393, Melexis), with a small size of 3 mm × 3 mm × 1 mm. In order to further ensure the flexibility of the device, an FPC board was used and designed into a serpentine shape. The schematic diagram of the FPC board is shown in Figure 6a. MLX90393 had an analog‐to‐digital converter inside, which can directly convert Hall voltage into a digital signal. The signal was then transmitted to the controller (Arduino Uno, Arduino SA) through inter‐integrated circuit (I2C). A multiplexer chip (TCA9548A, Texas Instruments) was used to send all eight channels of I2C data to the controller. The bluetooth serial port was used to send data in real‐time to a computer and a LabVIEW software was designed for signal processing such as Kalman filter, data display and data storage.

## 3 Results and Discussion

### 3.1 Basic Performance of the Sensor

**Figure 5:**
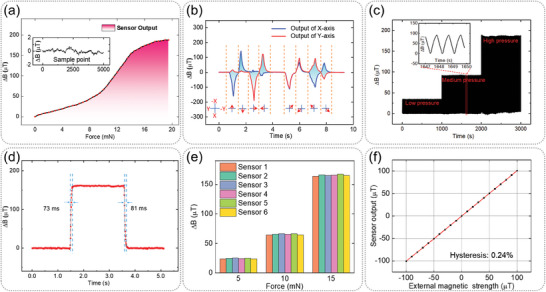
Performance results of the sensor. a) Response of the sensor to force. Inset: sensor noise without input. b) Response of the *X*‐axis and *Y*‐axis of the magnetic sensor to eight different directions of force. The eight red arrows below the curve indicate the force directions. c) Sensor output when the cilium is repeatedly bent under low, medium, and high pressure. Inset: output waveform of three bends. d) Test results of dynamic response time of our sensor. e) Consistency in the output of six sensors manufactured using the same process. f) The hysteresis of the magnetic sensor MLX90393.

For simplicity, a single cilium in the cilia array is first selected to characterize the basic performance of the tactile sensor, such as robustness and dynamic response time. The cilium is 500 µm in diameter and 5 mm in height. A precision testing platform is used for measurement, including a displacement guide rail (FUYU, FSL40XYZ‐L) with a step size as low as 1um, and a precision balance (Sartorius, BSA124S) with an accuracy of 1/10 000 grams. The balance is located below the displacement guide rail. The displacement guide rail can move vertically up and down, and our sensor with cilia is attached to the side. The schematic diagram is shown in Figure S1 (Supporting Information). A piece of acrylic block is placed on the balance. When the guide rail moves downward, the cilia will touch the acrylic block and then bend upward. The outputs of sensors and precision scales are collected using a LabVIEW program. **Figure** **5**a shows the response of the sensor to external force while the inset shows the noise of the sensor when there is no input. The noise range of the sensor is 1.3 µT. The response of the sensor to the external force can be divided into three sections. The initial section rises slowly, while the middle section rises faster and the latter section rises slowly. The reason why the middle section rises faster than the initial segment is that with the bending of the cilium, the distance between the whole cilium and the magnetic sensor is getting closer and closer, so the change rate of the magnetic field is changing faster and faster. The slow rise of the latter section is that the sensor output is close to saturation due to the limit of cilia bending. The sensitivity of the sensor in the initial stage, middle stage, and later stage is 6.63, 22.06, and 6.12 µT mN^−1^, respectively. According to the calculation of the initial section and noise, the resolution of the sensor to micro force is 0.2 mN. This performance is inferior to our previous research, but the working range of this sensor has increased to 19.5 mN from 80 µN. Therefore, it is much more suitable for robot applications such as object grasping.

Our sensor is able to distinguish the direction of external force because magnetic sensors used in this study are three‐axis magnetic sensor. The cilium is bent in eight directions, as shown in Figure 5b (the red arrows below the curve). The *X*‐axis and *Y*‐axis outputs of a magnetic sensor under the cilium are shown in Figure 5b. For −*X*, *X*, −*Y*, and *Y* directions, the output of the corresponding axis of the sensor changes significantly. Due to operational errors when bending the cilium, there are also small changes in the other direction, within an acceptable range. For oblique bending, there are both corresponding outputs in *X* and *Y* axes of the sensor.

Therefore, we can determine the arbitrary direction of bending by calculating the output size of different axes. Compared with tactile sensor that can only detect one direction, the ability to distinguish multiple directions is of great value in robot grasping and object sliding detection.

For flexible sensors, stability and dynamic response time are extremely important performances. First of all, a repeated bending experiment is conducted on our tactile sensor. The cilium is bent for 3000 times at a frequency of 1 Hz with three different forces in low, medium, and high levels, and results are shown in Figure 5c. It can be seen that under the forces of medium and low levels, the stability of cilia is very high. Under the bending force of high level, the output of cilia has some drift but the amplitude is not very large, which indicates that the sensor has good stability. We have conducted additional experiments to investigate the cycling response of the sensor along the *X* and −*X* directions (Figure S2, Supporting Information). The results indicate that there is no significant drift in the output of our sensor after multiple bending cycles. Furthermore, the output in the positive and negative directions of the *X*‐axis exhibits good consistency. Second, an experiment of rapid bending and release of a cilium is conducted to obtain the dynamic response time of the sensor. As shown in Figure 5d, the response and recovery time of the sensor is 73 and 81 ms, respectively. This is a normal performance in flexible devices, which can fully meet the needs of robot applications such as robot grasping.

Due to some differences in the manufacturing process, the performance of sensors from different batches may not be consistent. Therefore, consistency is a key performance parameter for flexible sensors. The same manufacturing process is used to produce six sensors (each with a single 5 mm cilium) for consistency testing. The outputs of each sensor under different forces are shown in Figure 5e. It can be seen that although there are indeed some differences in the outputs of the sensor, the trend of the output increasing with the increase of external force is similar. Last but not least, due to the use of magnetic sensors, the magnetic hysteresis of the sensor may have a detrimental impact on our tactile perception. Therefore, the magnetic hysteresis of the magnetic field sensor is also a performance parameter that needs attention. A Helmholtz coil enclosed within a seven‐layer magnetic shielding bucket (Figure S3, Supporting Information) is utilized to generate a magnetic field ranging from 0 to 100 µT, then to −100 µT, and finally back to 0 µT (limited by the Helmholtz coil's output). The output of the MLX90393, which is processed using a LabVIEW program, is presented in Figure 5f. As can be observed, the sensor exhibits almost no hysteresis, with a value of only 0.24%. Therefore, the hysteresis of magnetic sensors has almost no negative impact on tactile perception.

A comparison of our work with other related works is shown in **Table** **1**. It can be seen that our research has excellent force resolution. Despite the inferior resolution compared to our previous research,^[^ ^22^ ^]^ the working range has been greatly improved, making this sensor more suitable for practical applications in fields such as intelligent robots.

**Table 1:** Comparison of different magnetic tactile sensors.

Ref.	Resolution	Sensitivity	Range
[7]	10 mN	78 µV mN^−1^	4 N
[14]	0.71 mN	8.5–29.8 µT mN^−1^	3.4 N
[15]	0.33 mN	0.1 mV mN^−1^	7.8 mN
[17]	30 mN	—	1.9 N
[19]	6 mN	0.8 mV mN^−1^	55 mN
[22]	2.1 µN	0.63 mT mN^−1^	60 µN
This work	0.2 mN	6.63 µT mN^−1^	19.5 mN

“—” indicates that there is no relevant information in the paper.

### 3.2 Sliding Tactile Sensing

Grasping tasks play an important role in the development of intelligent robots. In the grasping process of robots, sliding detection can assist in adjusting the grasping force to ensure the stability of grasping.^[^ ^23^ ^]^ In addition, the occurrence of sliding can be largely avoided by detecting initial sliding, which is considerable in many grasping applications. For example, in surgical procedures, surgical forceps with sliding perception function are crucial for reducing surgical risks.

**Figure 6:**
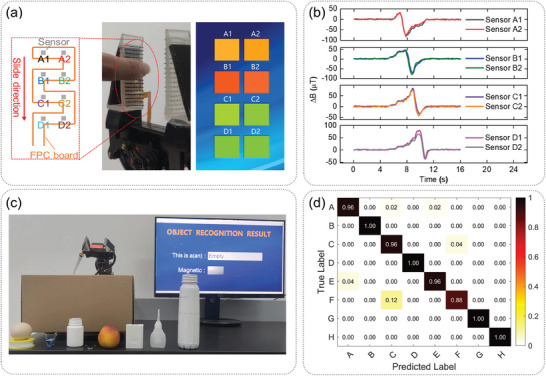
Application of our sensor on a robot flexible gripper. a) The left image shows eight magnetic sensors on a serpentine FPC board. The middle image is a photo of the robot flexible gripper. The right image shows a LabVIEW software, which can display the position and size of external force in real‐time. The darker the color, the greater the force. b) The output changes over time of eight sensors when a finger slides across the cilia array. c) Photo of object recognition with our flexible gripper. When an object is picked up by the gripper, the screen will display recognized results and whether it is magnetic. d) Confusion matrix of recognition results for eight different objects.

Compared with a single tactile sensor that can only perform single point measurement, our sensor array has the ability of sliding detection. The photo of the flexible gripper we designed is shown in the middle of **Figure** **6**a. The gripper is a hollow right triangle made of PDMS. The cilia array is located outside the long cathetus side of the triangle, and the magnetic sensor array is located inside the long cathetus side. Magnetic particles are also added to the hypotenuse of the triangle to enhance the detection ability to grip heavy objects. When the force on the gripper is small, or when the gripper picks up soft and light materials, the cilia array is first bent. On the contrary, when the force is large, the entire flexible gripper will deform. The distance between the hypotenuse and the FPC board will change, which will also cause changes in the sensor output. Therefore, this design can expand the working range of the flexible gripper. As shown in Figure 6a, eight magnetic sensors are distributed on the serpentine FPC board and are labeled as A1, A2, B1, B2, C1, C2, D1, and D2. A LabVIEW software is designed to display the position and size of external force. Figure 6b illustrates the test data derived from our sliding tactile perception experiment. As an object glides across the sensor surface, the utilization of a sensor array enables the immediate discernment of the prevailing sliding conditions by concurrently analyzing the distinct outputs from each sensor within the array. To illustrate, when an object undergoes sliding motions along the tactile gripper, as indicated by the red arrow in Figure 6a, the temporal evolution of sensor outputs is depicted in Figure 6b. Initially, the outputs of the top two sensors, A1 and A2, undergo simultaneous changes, succeeded by alterations in B1 and B2, and ultimately in D1 and D2. This sequential progression aligns with the direction of object sliding. The velocity of the object's sliding motion can be deduced from the temporal intervals between output changes and the spatial separation of sensors. Concurrently, the direction of the object's sliding motion can be ascertained by evaluating the order of output changes across distinct sensors. Movie S1 (Supporting Information) demonstrates the outstanding performance of our sensor in sliding tactile perception.

### 3.3 Object Recognition

Object recognition is a significant research field in pattern recognition. At present, the main method of object recognition is by using visual sensors such as cameras.^[^ ^24^, ^25^ ^]^ However, the recognition success rate of visual sensors is greatly affected by lighting conditions. In dark, narrow or blocked places, object recognition through vision is often not applicable. Tactile sensors are less restricted by environmental factors, and they can achieve accurate collection of object information in the environment where visual sensors are limited. At the same time, tactile sensors can also obtain object information of hardness and material.^[^ ^26^, ^27^ ^]^

The sensor proposed in this study can also realize object recognition through tactile sensing. As shown in Figure 6c, eight objects are selected for object recognition, and are labeled as letters A–H. C is an empty plastic bottle, while D is still this bottle, but an iron rod is placed inside it. Therefore, the appearance and shape of C and D are completely consistent, except for the internal magnetic differences. The flexible gripper grabs each object 100 times and data read by our sensor are saved and used for training. The training and testing datasets utilized are identical. Nevertheless, variations in the form or dimensions of objects can render recognition challenging for our sensors. This is primarily due to the tactile sensors' limited capacity to capture detailed information when compared to visual sensors, as they lack the spatial resolution and color discrimination capabilities of visual sensors. Despite the similarity between objects, slight positional variations may occur during training and testing. Fortunately, our sensor can effectively reduce the impact of these minor positional variations based on the shape and size of these objects. Subsequently, K‐nearest–neighbors algorithm is used for training and the training model is saved. Then, a serial port is used to read data of the sensor array in real time, and the saved model is called for object recognition. The recognition results are displayed in a LabVIEW software. The demonstration of object recognition is shown in Movie S2 (Supporting Information). The confusion matrix of the recognition results is shown in Figure 6d, and the overall recognition accuracy of our sensor reaches 97%. The success rate for objects C and D is 100%. This indicates that in addition to recognizing the shape and size of the object, our sensor can also identify whether there is magnetism inside the object. Tactile sensors based on piezoresistive, piezoelectric, and capacitive principles do not have this ability, which is of great value in lots of application fields such as ocean exploration, earthquake search and rescue.

## 4 Conclusions

In this work, a magnetic cilia array with tactile perception ability is developed, with a resolution of 0.2 mN in a working range of 19.5 mN. The large area of cilia array makes our sensor have the ability of sliding tactile sensation, which is considerable in various grasping applications. In addition, our sensor is able to detect the direction of external forces. Outstanding performances allow it to be used on a robot gripper. The flexible gripper not only has excellent tactile sensing function, but also can detect whether an object is slipping. By the help of machine learning algorithms, our sensor can realize object recognition with a success rate of 97%. In addition to recognizing the shape and size of an object, our sensor can identify whether there is magnetism inside the object. These excellent performances indicate that our sensor has extremely notable application value in multitudinous fields such as intelligent robots, advanced medical devices, ocean exploration, earthquake search, and rescue.

## Conflict of Interest

The authors declare no conflict of interest.

## Author Contributions

J.M. and J.C. designed the experiments. J.M. drafted the manuscript. Z.J. cooperated in the experiments and helped draft the manuscript, and J.C. checked it for final submission. All authors contributed to the article and approved the submitted version.

## Supporting information

Supporting Information

Supplemental Movie 1

Supplemental Movie 2

## Data Availability

The data that support the findings of this study are available from the corresponding author upon reasonable request.
