# Supplementary material for: Magnetic Tactile Sensor with Bionic Hair Array for Sliding Sensing and Object Recognition
Source: Adv Sci (Weinh). 2024 Jan 18;11(12):2306832. doi: 10.1002/advs.202306832 (PMC10966537; doi:10.1002/advs.202306832)
Supplement: Supplementary file 1 — Supporting Information [file ADVS-11-2306832-s001.pdf]

ADVANCED SCIENCE
Open Access

Supporting Information

for *Adv. Sci.*, DOI 10.1002/advs.202306832

Magnetic Tactile Sensor with Bionic Hair Array for Sliding Sensing and Object Recognition

*Jiandong Man, Zhenhu Jin and Jiamin Chen**

Supplementary Materials

Magnetic Tactile Sensor with Bionic Hair Array for Sliding Sensing and Object Recognition

Jiandong Man[1,2], Zhenhu Jin[1] and Jiamin Chen[1,2,3,]*

[1]State Key Laboratory of Transducer Technology, Aerospace Information Research Institute, Chinese Academy of Sciences, Beijing 100190, People's Republic of China
[2]School of Electronic, Electrical and Communication Engineering, University of Chinese Academy of Sciences; Beijing 100049, People's Republic of China
[3]College of Materials Sciences and Opto-Electronic Technology, University of Chinese Academy of Sciences, Beijing 100049, People's Republic of China

[*]Correspondence: Jiamin Chen (chenjm@aircas.ac.cn)

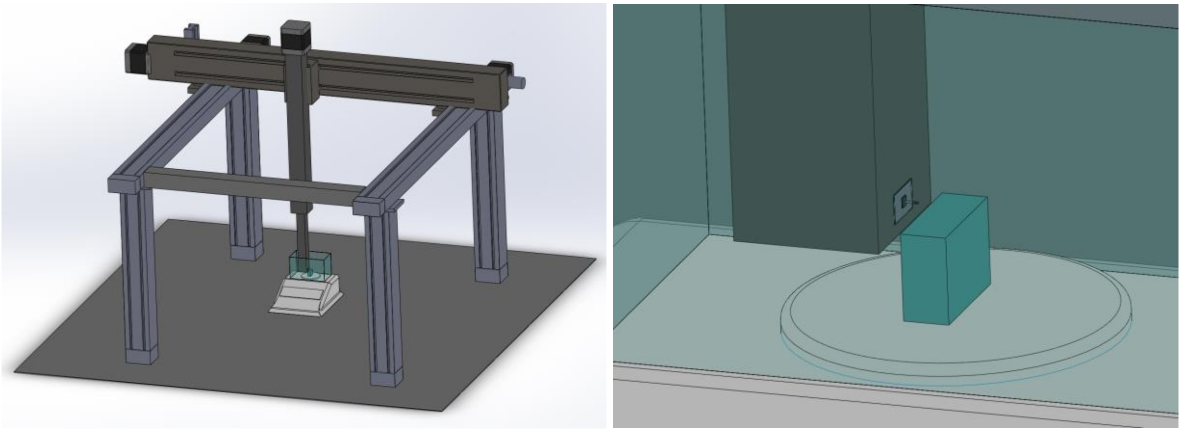

Figure. S1 Diagram of sensor testing system (Left) for small forces and local enlarged view (Right).

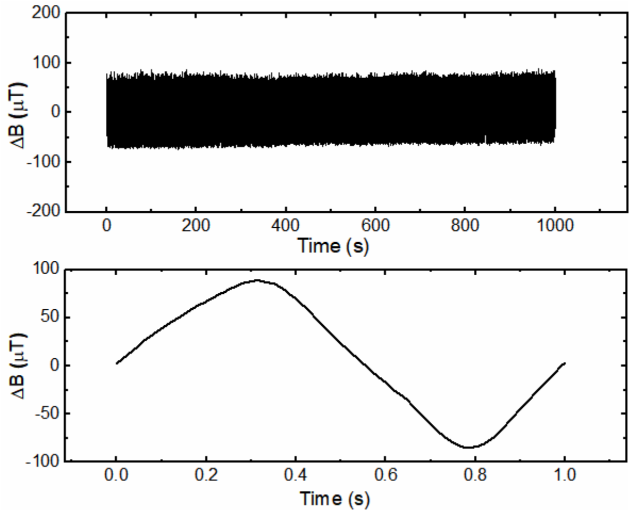

Figure. S2 The cycling response of the sensor along the X and -X directions (the image below shows one of the cycles).

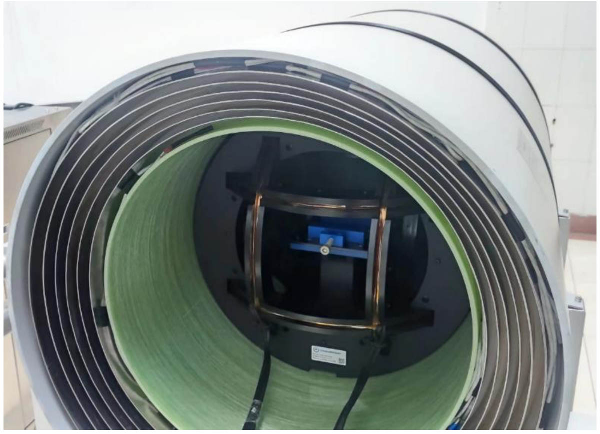

Figure. S3 Seven-layer magnetic shielding bucket and Helmholtz coil for performance testing of magnetic sensor MLX90393.
